# Gene transfer of GLT-1, a glial glutamate transporter, into the spinal cord by recombinant adenovirus attenuates inflammatory and neuropathic pain in rats

**DOI:** 10.1186/1744-8069-4-65

**Published:** 2008-12-24

**Authors:** Sanae Maeda, Ai Kawamoto, Yumi Yatani, Hisashi Shirakawa, Takayuki Nakagawa, Shuji Kaneko

**Affiliations:** 1 Department of Molecular Pharmacology, Graduate School of Pharmaceutical Sciences, Kyoto University, 46-29 Yoshida-Shimoadachi-cho, Sakyo-ku, Kyoto 606-8501, Japan

## Abstract

**Background:**

The glial glutamate transporter GLT-1 is abundantly expressed in astrocytes and is crucial for glutamate removal from the synaptic cleft. Decreases in glutamate uptake activity and expression of spinal glutamate transporters are reported in animal models of pathological pain. However, the lack of available specific inhibitors and/or activators for GLT-1 makes it difficult to determine the roles of spinal GLT-1 in inflammatory and neuropathic pain. In this study, we examined the effect of gene transfer of GLT-1 into the spinal cord with recombinant adenoviruses on the inflammatory and neuropathic pain in rats.

**Results:**

Intraspinal infusion of adenoviral vectors expressing the GLT-1 gene increased GLT-1 expression in the spinal cord 2–21 days after the infusion. Transgene expression was primarily localized to astrocytes. The spinal GLT-1 gene transfer had no effect on acute mechanical and thermal nociceptive responses in naive rats, whereas it significantly reduced the inflammatory mechanical hyperalgesia induced by hindlimb intraplantar injection of carrageenan/kaolin. Spinal GLT-1 gene transfer 7 days before partial sciatic nerve ligation recovered the extent of the spinal GLT-1 expression in the membrane fraction that was decreased following the nerve ligation, and prevented the induction of tactile allodynia. However, the partial sciatic nerve ligation-induced allodynia was not reversed when the adenoviruses were infused 7 or 14 days after the nerve ligation.

**Conclusion:**

These results suggest that overexpression of GLT-1 on astrocytes in the spinal cord by recombinant adenoviruses attenuates the induction, but not maintenance, of inflammatory and neuropathic pain, probably by preventing the induction of central sensitization, without affecting acute pain sensation. Upregulation or functional enhancement of spinal GLT-1 could be a novel strategy for the prevention of pathological pain.

## Background

Glutamate is the major excitatory neurotransmitter in the mammalian central nervous system. The spinal glutamatergic system plays a key role in normal pain transmission and in the induction of central sensitization, the neuronal plasticity underlying pathological pain at the spinal level. Glutamate release in the spinal dorsal horn is elicited following peripheral inflammation or nerve injury [1-5]. Excessive and prolonged stimulation of glutamate receptors, including *N*-methyl-D-aspartate (NMDA), α-amino-3-hydroxy-5-methyl-4-isoxazolepropionic acid/kainate, and metabotropic glutamate receptors, in the spinal dorsal horn neurons triggers the development of the central sensitization that generates and maintains inflammatory and neuropathic pain [6-8].

Extracellular glutamate released from nerve terminals is removed from the synaptic cleft via high-affinity, Na^+^-dependent glutamate transporters that surround excitatory synapses. This removal maintains the extracellular glutamate concentration in the physiological range, preventing the glutamate overexcitation and neurotoxicity that can occur under a variety of pathological conditions and modulating glutamate-mediated neuronal plasticity [9-11]. To date, five subtypes of glutamate transporters (excitatory amino acid transporters; EAATs) have been cloned and characterized in neurons (EAAC1/EAAT3, EAAT4, and EAAT5) and glial cells (GLT-1/EAAT2 and GLAST/EAAT1). Among these five subtypes, GLT-1 enriched in astrocytic processes appears to be the most abundant EAAT and may represent the predominant route for clearance of extracellular glutamate in the spinal cord [11]. Furthermore, astrocytes are able to specifically metabolize incorporated glutamate into glutamine with the enzyme glutamine synthetase [10]. The altered expression and function of glutamate transporters modulate glutamatergic signal transmission [9,12] and neuronal plasticity-based events such as long-term potentiation [13,14]. Indeed, the altered expression of glutamate transporters and altered glutamate uptake activity have been associated with neurodegenerative diseases, such as amyotrophic lateral sclerosis, epilepsy, and stroke [11,15-17]. In addition, several lines of evidence suggest that glutamate transporters have important roles in pathological pain [18]. Downregulation or functional deficiency of glutamate transporters in the spinal dorsal horn are associated with neuropathic pain following chronic constriction nerve injury [5,19,20], spinal nerve ligation [21] and spinal nerve transection [22], and hyperalgesia produced by paclitaxel (taxol), which is used for chemotherapy in cancer patients [23,24]. Pharmacological inhibition of glutamate transporters in the spinal cord leads to spontaneous nociceptive behaviors and hyperalgesia to mechanical and thermal nociceptive stimuli [25,26] by facilitating spinal glutamatergic synaptic activity [27]. In addition, riluzole, which increases glutamate uptake activity [28], attenuates neuropathic pain following chronic constriction nerve injury [19]. However, the lack of specific inhibitors and/or activators for glutamate transporter subtypes makes it difficult to determine the roles and subtypes of spinal glutamate transporters in pathological pain.

We previously constructed a recombinant adenovirus, termed Ad-GLT-1, to deliver the GLT-1 gene *in vitro *and *in vivo *[29,30]. In this study, to elucidate the role of a glial glutamate transporter, GLT-1, in the pathological pain, we examined the effect of recombinant adenovirus-mediated gene transfer of GLT-1 into the spinal cord on inflammatory and neuropathic pain in rats.

## Results

### Recombinant adenovirus-mediated GLT-1 expression in the spinal cord

**Figure 1:**
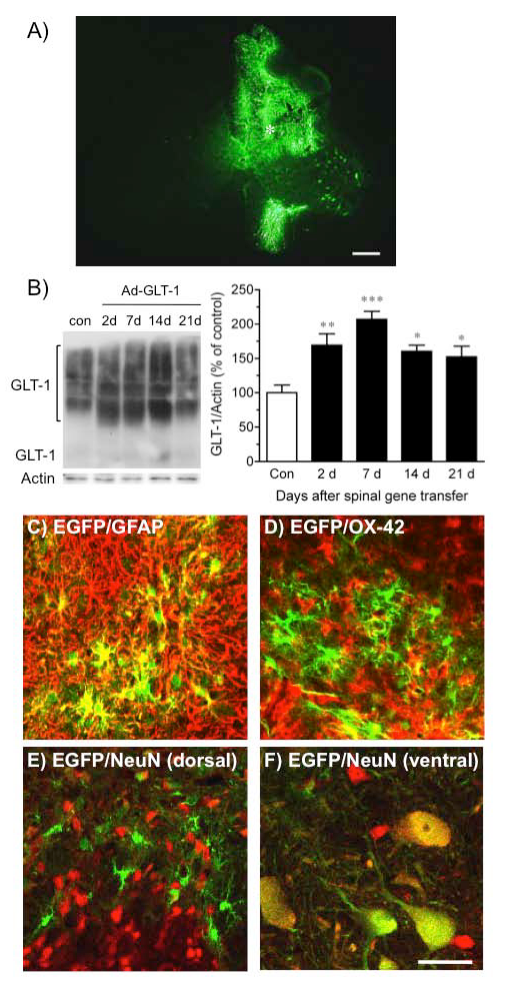
**GLT-1 and EGFP expression from recombinant adenoviruses in the spinal cord**. A) Representative fluorescence photomicrograph in the spinal cord 7 days after intraspinal infusion of Ad-EGFP. The asterisk indicates the infusion site. Scale bar = 300 μm. B, C) Western blot for GLT-1 in the ipsilateral spinal cord from rats 2, 7, 14 and 21 days after intraspinal infusions of Ad-GLT-1. B) Representative immunoblots show GLT-1 and actin immunoreactivity. C) Quantification of GLT-1 immunoreactivity. The signals were calculated by summing the monomer and multimer GLT-1 immunoreactivities. GLT-1 levels were normalized against the corresponding actin levels. The data present the means ± S.E.M. and are expressed as a percentage of the control (no intraspinal infusion). *P < 0.05, **P < 0.01, ***P < 0.001 vs control, *n *= 4. D, E, F) Immunofluorescence labelling of GFAP (D; red), a marker of astrocytes; OX-42 (E; red), a marker of microglia; and NeuN (F; red), a marker of neurons, with EGFP fluorescence (green) in the spinal cord 7 days after intraspinal infusion of Ad-EGFP. Most EGFP-positive cells are double-labelled (yellow) with GFAP. Some EGFP-positive cells are double-labelled with OX-42, but not with NeuN. Scale bar = 50 μm.

We first infused control adenoviruses, Ad-EGFP, into the spinal cord, which delivered the enhanced green fluorescence protein (EGFP) gene. Seven days after the intraspinal infusion of Ad-EGFP, fluorescence from EGFP expression was observed in the injected side of the dorsal horn and a part of the ventral horn of the spinal cord, with some transgene expression appearing within motor neurons (Fig. 1A). The infusion of adenoviruses was accompanied by minimal tissue damage and gliosis in the spinal cord, similar to that produced by infusion of phosphate-buffered saline (PBS; data not shown). In a Western blot, although immunoreactivities for endogenous GLT-1 were observed in the L4-L6 spinal cord, the expression of GLT-1 in the spinal cord was increased by intraspinal infusion of Ad-GLT-1, which delivered the GLT-1 gene (Fig. 1B). The significant increases in GLT-1 expression were observed between 2 and 21 days (*F *= 8.79, *P *< 0.001), which peaked between 2 and 14 days after the adenoviral infusion.

To examine the cell types that expressed the transgene, the sections from animals infused intraspinally with Ad-EGFP were immunostained with glial fibrillary acidic protein (GFAP), a marker of astrocytes, OX-42, a marker of microglia, or neuronal nuclei (NeuN), a marker of neurons. Throughout the dorsal horn of spinal cord, transgene expression was primarily localized to GFAP-positive astrocytes within the white and gray matter (Fig. 1C). A part of transgene expression was observed in OX-42-positive microglia (Fig. 1D), but not in NeuN-positive neurons (Fig. 1E). In the ventral horn, transgene expression was observed in motor neurons (Fig. 1F).

### Effects of spinal gene transfer of GLT-1 on acute nociceptive responses

**Figure 2:**
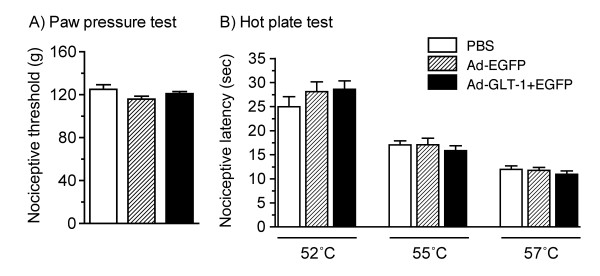
**Effects of spinal GLT-1 gene transfer on the acute mechanical and thermal nociceptive responses in naive rats**. Seven days after an intraspinal infusion of PBS (open bars), Ad-EGFP (hatched bars), or Ad-GLT-1 (closed bars), the mechanical nociceptive threshold (g) was measured in naive rats using the paw pressure test (A) and thermal nociceptive latency (sec) to 52, 55 and 57°C was measured using the hot plate test (B). Data are presented as the mean ± S.E.M., *n *= 12.

We examined the effects of PBS, Ad-EGFP, and Ad-GLT-1 on the acute mechanical and thermal nociceptive responses of naive rats 7 days after their intraspinal infusion. Intraspinal infusion of adenoviruses produced no apparent behavioral abnormalities and abnormal gait. In the paw pressure test, there was no significant difference in the mechanical nociceptive threshold among the groups infused intraspinally with PBS, Ad-EGFP, and Ad-GLT-1 (*F *= 2.04, *P *= 0.147; Fig. 2A). Similarly, there were no significant differences in the thermal nociceptive latency in the hot plate test (Fig. 2B) between the three groups at 52°C (*F *= 1.00, *P *= 0.379), 55°C (*F *= 0.40, *P *= 0.674), or 57°C (*F *= 0.60, *P *= 0.552).

### Effect of spinal gene transfer of GLT-1 on inflammatory hyperalgesia

**Figure 3:**
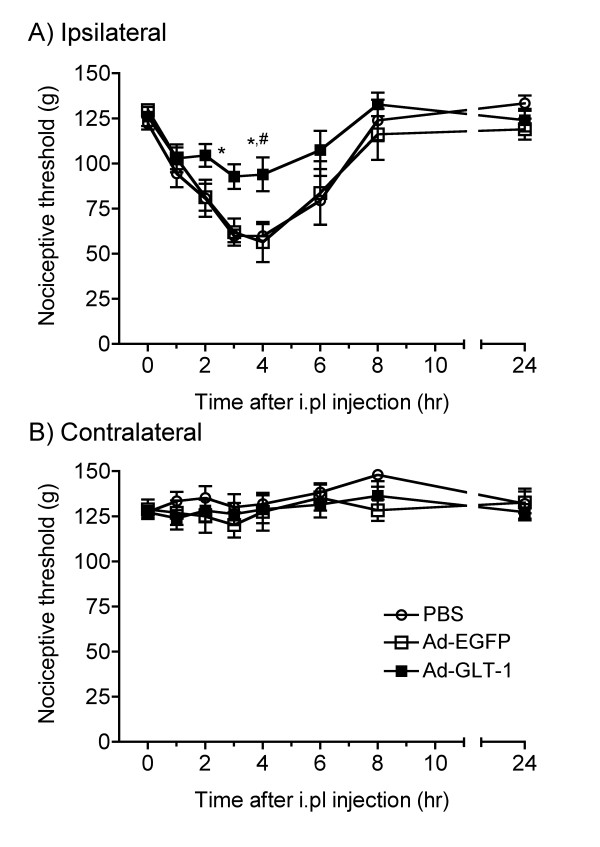
**Effect of spinal GLT-1 gene transfer on mechanical inflammatory hyperalgesia**. Seven days after an intraspinal infusion of PBS (open circle), Ad-EGFP (open square), or Ad-GLT-1 (closed square), the mechanical nociceptive thresholds of the ipsilateral (A) and contralateral (B) paws were measured in the paw pressure test following i.pl. injection of 2% carrageenan/kaolin at time 0. Data are presented as the mean ± S.E.M., *P < 0.05 vs PBS, ^#^*P *< 0.05 vs Ad-EGFP, *n *= 6–8.

We next infused PBS, Ad-EGFP, or Ad-GLT-1 into the spinal cord 7 days before intraplantar (i.pl.) injection of carrageenan/kaolin and examined the effect on inflammatory mechanical hyperalgesia in the paw pressure test (Fig. 3). In the animals that received an intraspinal infusion of PBS, i.pl. injection of carrageenan/kaolin decreased the mechanical nociceptive threshold of the ipsilateral hindpaw, which was maximal at 3–4 hours and diminished within 24 hours after the injection (Fig. 3A). The i.pl. carrageenan/kaolin did not change the mechanical nociceptive threshold of the contralateral hindpaw in the PBS group (Fig. 3B). Intraspinal infusion of Ad-EGFP also did not change the mechanical hyperalgesia in the ipsilateral paw in response to carrageenan/kaolin, similar to the group infused with PBS, but intraspinal infusion of Ad-GLT-1 significantly reduced the hyperalgesia compared with the groups infused with PBS and Ad-EGFP (*F*_2,144 _= 10.6, *P *< 0.001). Neither Ad-EGFP nor Ad-GLT-1 changed the mechanical nociceptive threshold in the contralateral paw compared with the group infused with PBS (*F*_2,144 _= 2.29, *P *= 0.105).

### Effect of spinal gene transfer of GLT-1 on neuropathic pain

**Figure 4:**
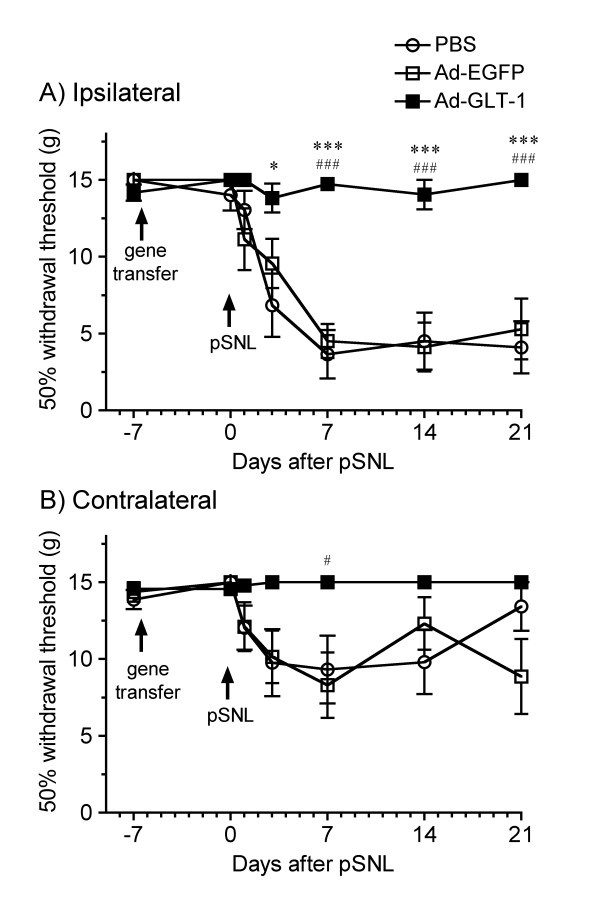
**Effect of spinal GLT-1 gene transfer on the induction of allodynia in the pSNL-induced neuropathic pain model**. PBS (open circle), Ad-EGFP (open square), or Ad-GLT-1 (closed square) was infused intraspinally (arrows in the graph). Seven days later (day 0), pSNL was performed (arrows) and 50% withdrawal thresholds of the ipsilateral (A) and contralateral (B) paws were measured with the von Frey filament test on the indicated days. Data are presented as the mean ± S.E.M. *P < 0.05, ***P < 0.001 vs PBS; ^#^*P *< 0.05, ^###^*P *< 0.001 vs Ad-EGFP, *n *= 6–9.

**Figure 5:**
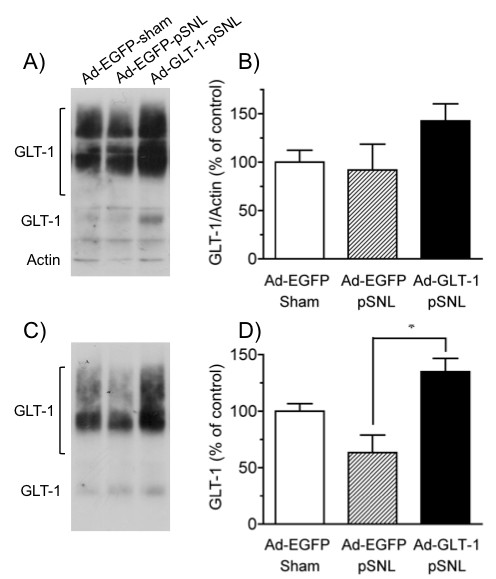
**Effect of spinal GLT-1 gene transfer on the spinal GLT-1 expression in the total and membrane fraction following pSNL**. Seven days after Ad-EGFP or Ad-GLT-1 was infused intraspinally, pSNL or sham surgery was performed. Seven days later (14 days after the intraspinal infusion), the GLT-1 expression in the total (A, B) and membrane (C, D) fractions obtained from ipsilateral spinal cord was assessed by Western blot. A, C) Representative immunoblots show GLT-1 and actin immunoreactivity in total and membrane fractions. No actin band was observed in the membrane fraction. B, D) Quantification of GLT-1 immunoreactivity in the total and membrane fractions. The signals were calculated by summing the monomer and multimer GLT-1 immunoreactivities. Total GLT-1 levels were normalized against the corresponding actin levels. The data present the means ± S.E.M. and are expressed as a percentage of the control (intraspinal infusion of Ad-EGFP and sham surgery) for each fraction. *P < 0.05, *n *= 3–4.

To investigate the effect of spinal gene transfer of GLT-1 on the induction of neuropathic pain following partial sciatic nerve ligation (pSNL), we performed pSNL 7 days after the intraspinal infusion of the adenoviruses (Fig. 4). In the animals that received PBS, pSNL decreased the 50% withdrawal threshold of the ipsilateral hindpaw to tactile stimulation with von Frey filaments, a decrease that lasted for at least 21 days. The contralateral hindpaw also showed substantial tactile allodynia, although it was weaker than that in the ipsilateral hindpaw. Intraspinal infusion of Ad-EGFP did not change the tactile allodynia in the ipsilateral and contralateral hindpaw, which was similar to that in the group infused with PBS. In the group infused with Ad-GLT-1, the pSNL-induced tactile allodynia was completely prevented, a significant change from the results in the PBS- and Ad-EGFP-infused groups (*F*_2,168 _= 35.1, *P *< 0.001). Intraspinal infusion of Ad-GLT-1 similarly significantly prevented the pSNL-induced allodynia in the contralateral hindpaw (*F*_2,168 _= 9.96, *P *< 0.001). The GLT-1 expression in the total and membrane fractions obtained from ipsilateral spinal cord was examined by Western blot (Fig. 5). In the animals infused with Ad-EGFP, total amount of GLT-1 protein was not changed, while the GLT-1 level in the membrane fraction decreased at 7 days following pSNL (14 days after intraspinal infusion). Intraspinal infusion of Ad-GLT-1 to pSNL-treated animals increased the GLT-1 expression in the total and membrane fractions. The recovery of the extent of GLT-1 expression was significant, compared with pSNL-treated animals infused with Ad-EGFP.

**Figure 6:**
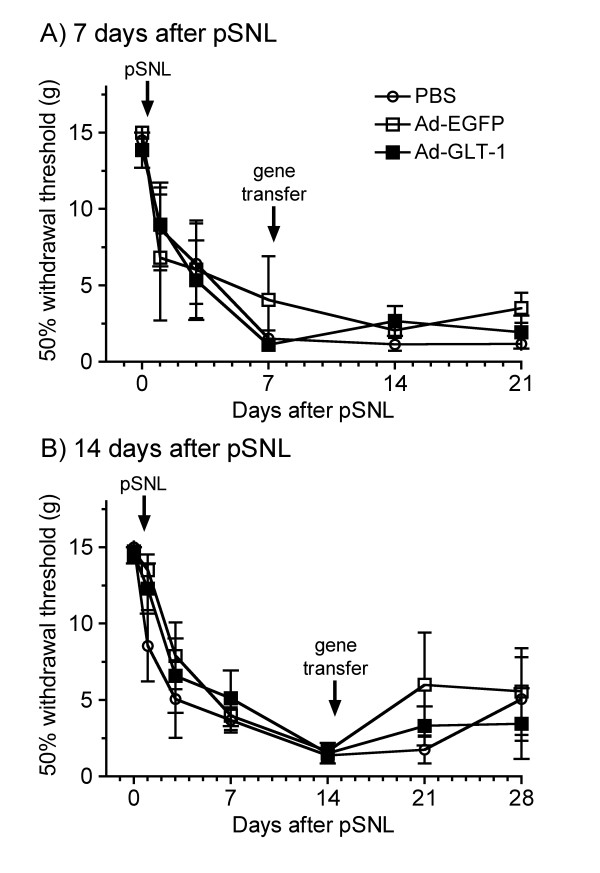
**Effect of spinal GLT-1 gene transfer on the maintenance of allodynia in the pSNL-induced neuropathic pain model**. PBS (open circle), Ad-EGFP (open square), or Ad-GLT-1 (closed square) was intraspinally infused 7 (A) or 14 (B) days after pSNL (arrows in the graph). 50% withdrawal thresholds of ipsilateral paw were measured with the von Frey filament test on the indicated days following pSNL at day 0. Data are presented as the mean ± S.E.M., *n *= 4–6.

Finally, to investigate the effect of spinal gene transfer of GLT-1 on the maintenance of neuropathic pain, we intraspinally infused PBS or recombinant adenoviruses 7 or 14 days after pSNL (Fig. 6). However, intraspinal infusion of Ad-GLT-1 had no effect on the established tactile allodynia in the ipsilateral hindpaw at 7 or 14 days following pSNL, compared with the PBS- and Ad-EGFP-infused groups (*F*_2,70 _= 0.29, *P *= 0.750 and *F*_2,91 _= 1.73, *P *= 0.183, respectively).

## Discussion

We previously reported that an infusion of Ad-GLT-1 into specific brain areas efficiently increased GLT-1 expression at 2 and 5 days, and this expression remained stable up to 8 days after the infusion [29,30]. In the present study, we confirmed that intraspinal infusion of the recombinant adenovirus successfully transferred the GLT-1 gene into the spinal cord surrounding the infusion site between 2 and 21 days. Our previous finding showed that infusion of the recombinant adenovirus was accompanied by minimal tissue damage and had no effect on the immunoreactivity of GFAP surrounding the infusion site [29,30], suggesting that no toxicity or gliosis resulted from the adenoviral infection. Although an adenovirus transferred the LacZ gene efficiently into both neurons and glial cells [31], the majority of cells with adenovirus-mediated gene transfer in the dorsal horn were astrocytes when the adenoviruses were infused into the spinal cord, consistent with previous reports [32]. Because GLT-1 is expressed mainly in astrocytes, the adenovirus-mediated expression system seemed likely to be suitable for the present study. In the ventral horn, the transgene expression was observed in astrocytes and motor neurons. Of all the neurons within the spinal cord, motor neurons have been shown to efficiently uptake and express recombinant adenoviruses [32,33]. Although we did not determine the influence of GLT-1 gene transfer in motor neurons in this study, at least, it did not induce behavioral abnormalities and abnormal gait, and had no effect on the escape behaviors from the mechanical and thermal nociceptive stimuli.

Using the recombinant adenoviruses, we showed that gene transfer of GLT-1 into the spinal cord had no effect on the acute nociceptive responses to mechanical and thermal stimuli in naive rats. These data are supported by previous studies using glutamate transporter activators such as MS-153 [34] and riluzole [4,19]. In contrast, intrathecal injection of glutamate transporter inhibitors into naive animals elevates the spinal extracellular glutamate level and produces spontaneous nociceptive behaviors and hyperalgesia [25-27]. These results suggest that maintaining a low extracellular glutamate level following an increase in spinal GLT-1 protein does not affect acute spinal pain transmission under normal conditions. In addition, the important finding in this experiment is to show no evidence that adenoviral infection in the spinal cord influenced the basal nociceptive threshold.

Here we showed that spinal gene transfer of GLT-1 reduced i.pl. carrageenan/kaolin-induced inflammatory mechanical hyperalgesia and pSNL-induced tactile allodynia, when the spinal infusion of the adenoviruses was performed 7 days before i.pl. injection of carrageenan/kaolin and pSNL surgery, respectively. Furthermore, it recovered the extent of the spinal GLT-1 expression in the membrane fraction that was decreased at 7 days following pSNL. These results suggest that adenovirus-mediated overexpression of GLT-1 in the spinal cord prevents the induction of inflammatory and neuropathic pain. Consistent with the present data, riluzole, which reduces extracellular glutamate by activating glutamate transporters [28], inhibits the induction of inflammatory and neuropathic pain [4,19]. Glutamate release from primary afferent neurons in the spinal dorsal horn is enhanced by peripheral inflammation and nerve injury [1-5]. Excessive and prolonged activation of spinal glutamate receptors and subsequent intracellular adaptation in the postsynaptic dorsal horn neurons lead to a prolonged increase in neuronal excitability, called central sensitization, which produces pathological pain [7,8]. Indeed, inhibition of glutamate release by anticonvulsant agents attenuates the induction of hyperalgesia following peripheral inflammation or nerve injury [4,35]. Our results suggest that the increase of glutamate uptake activity following overexpression of spinal GLT-1 protein decreases the excessive extracellular glutamate level; this in turn inhibits the generation of neuronal plasticity related to central sensitization following peripheral inflammation and nerve injury.

In the present pSNL model, tactile allodynia was produced also in the contralateral to the nerve injury site, called mirror-image pain. It is considered that mirror-image pain arises from altered contralateral spinal processing of incoming sensory information [36]. The present study showed that contralateral allodynia was also prevented by the unilateral gene transfer of GLT-1 into the ipsilateral spinal cord, although the unilateral gene transfer did not spread to the contralateral spinal cord. Milligan et al. demonstrated that mirror-image pain may be due to spinal glial activation and release of proinflammatory cytokines, which may spread and reach the contralateral spinal cord to generate sensitization of the contralateral dorsal horn neurons [37]. The spinal glial activation following peripheral nerve injury is dependent on spinal NMDA receptor activation [38,39]. Consequently, it is conceivable that the ipsilateral gene transfer of GLT-1 may attenuate the activation of glial cells and the release of proinflammatory cytokines in the ipsilateral spinal cord by inhibiting a glutamate-dependent pathway, which has the effect of preventing the induction of sensitization of contralateral, as well as ipsilateral, dorsal horn neurons.

In contrast to the ability of exogenous GLT-1 to prevent the induction of pathological pain, when the spinal infusion of the adenoviruses was performed 7 or 14 days after pSNL, spinal gene transfer of GLT-1 did not reverse the established allodynia. Many previous studies have shown that the expression of GLT-1 and glutamate uptake activity are decreased 7–14 days after nerve injury [5,19,20,22]. Similarly, we found a decrease in GLT-1 expression in the membrane fraction of the spinal cord at 7 days following pSNL, although the total amount of GLT-1 protein was not changed in the present pSNL-induced neuropathic pain model in contrast to previous reports using other neuropathic pain models [19,22]. The spinal glutamatergic system contributes to not only the induction of pathological pain, but also to its maintenance [6,40,41]. Sung et al. reported that riluzole twice daily for 4 days beginning on postoperative day 5 gradually reversed the maintenance of thermal hyperalgesia and mechanical allodynia after chronic constriction nerve injury [19]. However, riluzole has multiple actions in addition to activating glutamate transporters, including blockade of sodium channel α-subunits, glutamate receptors, and γ-aminobutyric acid uptake and the stabilization of voltage-gated ion channels [42]. The inhibitory effect of riluzole on the maintenance of neuropathic pain may therefore be due to actions other than the activation of glutamate transporters. Our present findings suggest that the expression of GLT-1 in the spinal cord plays little role in the maintenance of neuropathic pain, at least in the present conditions. Otherwise, the expression of other EAAT subtypes, GLAST and EAAC1, was also decreased in the spinal cord of neuropathic pain model animals [19,20,22-24]. Reduction of glutamate uptake activity via GLAST and EAAC1 may contribute to the maintenance of neuropathic pain.

An accumulating amount of evidence suggests that spinal astrocytes, as well as microglia, contribute to pathological pain [43,44]. Early studies indicated that spinal astrocytes are activated in diverse models of pathological pain [37,45,46], and blocking the activation and function of spinal astrocytes prevents and reverses hyperalgesia and allodynia [37,47,48]. Because GLT-1 is expressed mainly in astrocytes, the present findings may further support the importance of spinal astrocytes in pathological pain.

## Conclusion

The present study showed that recombinant adenovirus-mediated gene transfer of GLT-1 into the spinal cord reduces the induction of inflammatory and neuropathic pain without affecting basal nociceptive responses. These findings support the idea that altered expression or function of GLT-1 in the spinal cord contributes to the pathogenesis of inflammatory and neuropathic pain. Therefore, upregulation or functional enhancement of spinal GLT-1 may prevent the induction and progression of pathological pain. Glutamate receptor antagonists are effective in reducing pathological pain in animal models and clinical settings, but their usefulness is limited by adverse side-effects [49]. Further studies to delineate the roles of spinal glutamate transporters in pathological pain states might lead to better strategies for the prevention of pathological pain.

## Methods

### Animals

Male Sprague-Dawley rats initially weighting 180–220 g were used. They were kept at a constant ambient temperature of 24 ± 1°C under a 12-hr light/dark cycle and provided free access to food and water. The rats were individually housed in plastic cages with wood-chip bedding for at least 1 day before surgery. All experimental procedures were approved by the Kyoto University Animal Experimentation Committee and complied with the recommendations of the International Association for the Study of Pain [50].

### Construction of recombinant adenoviruses

Construction of the recombinant adenoviruses was described previously [29]. Briefly, fragments of rat GLT-1 and EGFP cDNAs were subcloned, respectively, into multiple cloning sites 1 and 2 of the vector pIRES (Clontech, Palo Alto, CA, USA). A 3.5-kb fragment containing the GLT-1-internal ribosome entry site-EGFP sequence was then subcloned into the cosmid vector pAxCAwt (Takara, Kyoto, Japan), which contains the CAG promoter (cytomegalovirus enhancer and β-actin promoter) and the rabbit β-globin polyadenylation signal [51]. For the control adenovirus, only the EGFP fragment was subcloned into pAxCAwt. The recombinant adenoviruses were generated using an Adenovirus Expression Vector kit (Takara) according to the manufacturer's instructions and named Ad-GLT-1 and Ad-EGFP. They were propagated in HEK293 cells and purified using the Viraprep Adenovirus Purification Kit (Virapur, San Diego, CA). The titers of Ad-GLT-1 and Ad-EGFP were 5.6 × 10^8 ^to 1.0 × 10^10 ^plaque-forming units/ml.

### Intraspinal infusions of recombinant adenoviruses

Infusions of the recombinant adenoviruses into the spinal cord were performed as reported previously [32]. Briefly, animals that were anesthetized with pentobarbital sodium (50 mg/kg, i.p.) underwent hemilaminectomies at the L1-L4 vertebral segments. Intraspinal infusion was performed unilaterally on the right side. After exposure of the spinal cord, each animal received four injections (0.4 μl; 0.8 mm apart and 0.5 mm deep) of PBS or individual adenoviral vectors along the L4-L5 dorsal root entry zone using a beveled glass micropipette. In this procedure, the tip of glass micropipette reached lamina IV-VI of the spinal cord. The dorsal muscle and skin were then sutured. The animals were placed on a 37°C heat pad and monitored continually until recovery from the effects of the anesthetic.

### Tissue preparation and Western blot

Animals were rapidly sacrificed by decapitation. The L4-L6 lumber spinal cord was rapidly removed, and cut into a left and right half from the ventral midline. Then, the right lumbar spinal cord segments were immediately frozen in liquid nitrogen and stored at -80°C until use. The segments were homogenized with a polytron homogenizer in ice-cold 20 mM Tris buffer (pH 7.5) containing 0.32 M sucrose, 2 mM ethylenediaminetetraacetic acid (EDTA), 0.5 mM ethyleneglycol-bis (β-aminoethyl)-N,N,N',N'-tetraacetic acid (EGTA) and a cocktail of protease inhibitors (Calbiochem, San Diego, CA). Some of the homogenate was separated and saved for western blot as total fraction. Remains were centrifuged (1,000 × g, 5 min, 4°C) to remove nuclear debris. The resulting supernatants were centrifuged (18,000 × g, 30 min, 4°C) to obtain the synaptosomal pellet. The synaptosomal pellets were resuspended in ice cold water containing p a cocktail of protease inhibitors to lyse the synaptosomal membranes for 45 min on ice. The lysed synaptosomal sample was centrifuged (40,000 × g, 15 min, 4°C) to pellet synaptosomal membranes, which was then resuspended in 20 mM Tris buffer (pH 7.4) containing 2 mM EDTA, 0.5 mM EGTA, 1% Triton X-100 and a cocktail of protease inhibitors, and saved for western blot (membrane fraction). The protein concentrations were measured. Samples were stored at -20°C until the Western blot analysis.

Aliquots of protein sample (2 μg) prepared from total or membrane fractions were diluted with an equal volume of sample buffer (124 mM Tris-HCl (pH 7.5), 4% sodium dodecyl sulphate (SDS), 10% glycerol, 4% 2-mercaptoethanol and 0.02% bromophenol blue), subjected to SDS-polyacrylamide gel electrophoresis, and electrophoretically transferred onto polyvinylidene fluoride membranes (Millipore, Bedford, MA). Blots were blocked for 1 hr with 10% blocking reagent (Blocking-One, Nacalai tesque, Kyoto, Japan) in Tris-buffered saline (pH 7.5) containing 0.1% Tween-20, and then incubated with goat anti-GLT-1 (1:2,000; Santa Cruz Biotechnology, Santa Cruz, CA) and goat anti-actin (1:20,000; Santa Cruz Biotechnology) for 1 hr at room temperature. Then, the blots were incubated with horseradish peroxidase-conjugated donkey anti-goat IgG (1:25,000, Jackson ImmunoResearch Laboratories, West Grove, PA) for 1 hr at room temperature. The immunoreactive proteins were detected with an enhanced chemiluminescence kit (Amersham Biosciences) according to the manufacturer's instructions, and visualized by exposure to X-ray film. For the quantification of Western signals, the signal for each lane was calculated by summing the (area × [density-background]) measurement for the monomer and multimer GLT-1 bands with a computer-assisted imaging analysis system (Image J 1.39).

### Histology and immunohistochemistry

Animals were deeply anesthetized with sodium pentobarbital (50 mg/kg, intraperitoneally) and perfused transcardially through the ascending aorta with 0.1 M PBS (pH 7.4), immediately followed by 4% paraformaldehyde in 0.1 M phosphate buffer. The L4-L6 lumbar spinal cord was removed, post-fixed in the same fixative for 3 hr, cryoprotected with 15% sucrose in 0.1 M phosphate buffer overnight at 4°C, and then frozen in liquid nitrogen. Coronal sections (30 μm) were thaw-mounted onto MAS coat slide glasses (Matsunami, Osaka, Japan).

For histological examination of EGFP fluorescence, each section was examined by fluorescent microscopy for EGFP. For immunohistochemical examination of cell markers with EGFP fluorescence, the sections were gently washed three times (10 min each) in PBS, and then permeabilized and blocked at room temperature for 1 hr in 4% normal goat serum in PBS containing 0.1% Triton X-100. For immunofluorescence imaging of the marker proteins for neurons, astrocyte and microglia, spinal the spinal sections were incubated overnight at 4°C with mouse anti-NeuN (1:100, Chemicon, Temecula, CA), mouse anti-GFAP (1:100, Sigma, St. Louis, MO) or mouse anti-OX-42 (1:100, Serotic, Ltd., Oxford, UK) monoclonal antibody in PBS containing 0.1% Triton X-100 and 4% normal goat serum. The sections were washed three times in PBS, and incubated with Alexa Fluoro 568-labeled goat anti-mouse IgG antibody (1:200; Molecular Probes) in PBS with 0.1% Triton X-100 and 4% normal goat serum for 1 hr at room temperature. Images were constructed by measuring the fluorescence signal using a Nikon Diaphot 200 microscope equipped with a laser scanning confocal imaging system (MRC-1024 system; Bio-Rad Laboratories, Hercules, CA) with excitation lines of 488 and 568 nm.

### Inflammatory and neuropathic pain models

#### Inflammatory pain model

Acute inflammation was produced by subcutaneous injection with a mixture of 2% λ-carrageenan and 2% kaolin dissolved in saline (0.1 ml) into the plantar region of the right hind paw, as previously described [52].

#### Neuropathic pain model

pSNL, a well-characterized rat model of neuropathic pain, was performed as previously described [53]. Briefly, under diethylether anesthesia, a skin incision was made, and the right sciatic nerve was exposed just distal to the branch leading to the posterior biceps femoris/semitendinosus muscles. The 1/3–1/2 dorsal section of the sciatic nerve was ligated tightly with 7-0 silk suture. The wound was closed by suturing the muscle and skin layers. After recovering from the anesthesia, almost all animals showed guarding of the hind paw, but none engaged in autotomy.

### Behavioral tests

#### Hot plate test

Thermal nociception was evaluated by the hot plate test using a hot plate analgesy meter (Ugo Basile, Milan, Italy). The animal was placed on a plate heated to 52, 55, or 57°C and the latency to licking a hindpaw or jumping was measured. The cut-off time was 60 sec to prevent tissue damage.

#### Paw pressure test

For assessing the mechanical nociceptive threshold, the paw pressure test was performed using an analgesimeter with a cuneate piston (Ugo Basile). The piston was loaded at a rate of 16 g/sec. The pressure-elicited paw-withdrawal behavior was determined as a nociceptive threshold. The procedure for paw pressure testing was carried out 3 times per day for habituation. After 2 days of habituation, the threshold was measured following three additional habituation procedures and the value was taken as a control.

#### Von Frey filament test

Tactile allodynia was measured by the up-down method as described previously [54]. Animals were individually placed on a wire mesh floor and acclimatized to the environment for at least 30 minutes. After acclimatization, the tactile stimulus was applied to the middle plantar surface of the paw by placing one of a series of von Frey filaments (0.4, 0.6. 1.4, 2.0, 4.0, 6.0, 8.0, 10.0, 15.0 g) perpendicular to the surface of the paw. The testing was initiated at 2.0 g. In the absence of a paw withdrawal response to the initially selected filament, a stronger stimulus was presented; in the event of paw withdrawal, the next weaker stimulus was chosen. Four additional responses were observed after the first withdrawal response, and the 50% withdrawal threshold was calculated [55]. In cases where continuous positive or negative responses were observed to the exhaustion of the stimulus set, values of 15.00 g and 0.4 g were assigned, respectively.

### Statistical analysis

Data are expressed as means ± S.E.M. In the behavioral tests and Western blot analyses, the statistical significance was calculated using one-way and two-way analysis of variance, followed by the Bonferroni *post hoc *test. Differences with *P *< 0.05 were considered significant.

## Abbreviations

EAAT: excitatory amino acid (glutamate) transporters; EDTA: ethylenediaminetetraacetic acid; EGFP: enhanced green fluorescence protein; EGTA: ethyleneglycol-bis (β-aminoethyl)-N,N,N',N'-tetraacetic acid; i.pl.: intraplantar; GFAP: glial fibrillary acidic protein; NeuN: neuronal nuclei; NMDA:*N*-methyl-D-aspartate; PBS: phosphate-buffered saline; pSNL: partial sciatic nerve ligation; SDS: sodium dodecyl sulphate.

## Competing interests

The authors declare that they have no competing interests.

## Authors' contributions

SM carried out all experiments, performed the statistical analysis, and drafted the manuscript. AK assisted with the behavioral experiments. YY generated the recombinant adenoviruses. HS participated in the data analysis and interpretation. TN contributed to the design of the study, the behavioral experiments, the data analysis and interpretation, and drafting and critical review the manuscript. SK participated in the data analysis and interpretation and contributed to drafting and final review of the manuscript.
